# Load Position and Weight Classification during Carrying Gait Using Wearable Inertial and Electromyographic Sensors

**DOI:** 10.3390/s20174963

**Published:** 2020-09-02

**Authors:** Maja Goršič, Boyi Dai, Domen Novak

**Affiliations:** 1 Department of Electrical and Computer Engineering, University of Wyoming, Laramie, WY 82071, USA; mgorsic@uwyo.edu; 2 Division of Kinesiology and Health, University of Wyoming, Laramie, WY 82071, USA; bdai@uwyo.edu

**Keywords:** carrying, classification, electromyography, gait, inertial measurement units, supervised machine learning, wearable sensors

## Abstract

Lifting and carrying heavy objects is a major aspect of physically intensive jobs. Wearable sensors have previously been used to classify different ways of picking up an object, but have seen only limited use for automatic classification of load position and weight while a person is walking and carrying an object. In this proof-of-concept study, we thus used wearable inertial and electromyographic sensors for offline classification of different load positions (frontal vs. unilateral vs. bilateral side loads) and weights during gait. Ten participants performed 19 different carrying trials each while wearing the sensors, and data from these trials were used to train and evaluate classification algorithms based on supervised machine learning. The algorithms differentiated between frontal and other loads (side/none) with an accuracy of 100%, between frontal vs. unilateral side load vs. bilateral side load with an accuracy of 96.1%, and between different load asymmetry levels with accuracies of 75–79%. While the study is limited by a lack of electromyographic sensors on the arms and a limited number of load positions/weights, it shows that wearable sensors can differentiate between different load positions and weights during gait with high accuracy. In the future, such approaches could be used to control assistive devices or for long-term worker monitoring in physically demanding occupations.

## 1. Introduction

Lifting and carrying heavy objects is a major aspect of physically intensive jobs such as construction, and is the cause of many work-related injuries [1]. One potential way to reduce such injuries would be through active and passive (motorized or nonmotorized) assistive devices such as exoskeletons that can support the body during lifting, reducing load on the trunk [2,3,4,5,6]. However, to intelligently support the wearer (by either applying forces to the wearer using motors [4] or by modifying their own structure for better support [2,5]), such devices need to be aware of the user’s desired intentions: for example, when the user starts lifting an object, how they grip it, and how they hold it while carrying it. This can be achieved using sensors that are either built into the device or worn by the human.

The two most common types of human-worn sensors used with assistive devices are inertial measurement units (IMUs), which measure kinematics with a combination of accelerometer and gyroscope (and optionally magnetometer and/or barometer), and electromyographic (EMG) sensors, which noninvasively measure the electrical activity of human muscles. Both IMUs and EMG sensors were previously extensively used to, e.g., control active lower limb prostheses [7] and classify different reaching motions [8], and could thus be applied to lifting and carrying as well. The promise of such sensors is supported by previous studies that used optical tracking cameras, which measure similar quantities as IMUs (kinematics); such cameras can, for example, quantify back load [9], identify risky postures [10], differentiate between novice and expert load lifters [11], differentiate between different load weights [12], and differentiate between holding loads in front or at the sides of the body [13].

To be used with assistive devices, wearable devices would need to detect the beginning and end of a lifting and carrying process as well as characterize how the process is performed. For example, is the person lifting by squatting low to the ground or by bending at the back? Once the object has been picked up, does the person carry it in front of their body with both hands, or at the side with one hand? Differentiating between such behaviors would allow assistive devices to better support the user by, e.g., knowing when to begin assisting and how to provide support appropriate for the user’s current activity, similarly to existing control approaches in technologies such as lower limb prostheses and exoskeletons [7,14,15,16,17,18].

If we separately consider lifting behavior (the process of picking up an object) and carrying behavior (walking while holding an object), wearable sensors have already been combined with pattern recognition algorithms to automatically detect and characterize lifts. For example, Brandt et al. [19] developed a method to classify a lift as high or low risk using a combination of IMUs and EMG sensors, and achieved an accuracy of 78.1% using a subject-specific (personalized) classifier. Similarly, O’Reilly et al. [20] classified deadlifts as ‘good’ or ‘bad’ using five IMUs on the back, and achieved an accuracy of 93% with a subject-specific classifier and 75% with a subject-nonspecific (generic) classifier. Finally, in our own previous study [21], we developed algorithms to detect the start and end of a lift as well as to determine multiple lift characteristics (stooping vs. squatting, vertical and horizontal movement) using 17 IMUs, and achieved high accuracies. Thus, we believe that, at this point, sensor-based pattern recognition methods are well-developed enough to automatically characterize lifting behavior.

Conversely, carrying behavior has been studied with wearable sensors in significantly less detail. While camera-based studies have shown significant differences between different load locations and weights [22,23,24,25,26,27,28], to the best of our knowledge only four studies have attempted to automatically classify different load carriage types with wearable sensors, which would be the prerequisite for control of assistive devices. Benocci et al. [29] used four IMUs and a k-nearest-neighbor classifier to differentiate among six backpack positions (all with the same weight): none, on both shoulders, on only left shoulder, on only right shoulder, in right hand, and in left hand; they achieved an overall classification accuracy of 96.7%. Yang et al. [30] used a single IMU and a bidirectional long short-term memory algorithm to study three different classification problems in a scenario with four different load weights (all held in front of the chest with both hands); they were able to differentiate between no-weight and weight conditions with an accuracy of 98.6%, between no-weight, low weight and high weight with an accuracy of 86.4%, and between no-weight, low weight, medium weight, and high weight with an accuracy of 76.4%. Lim and D’Souza [31] used four IMUs and a random forest classifier to differentiate among nine conditions consisting of a no-weight condition as well as all combinations of four load positions (one-handed left, one-handed right, two-handed with one side load in each hand, two-handed with one larger load in front of the chest) and two load weights; they achieved a load position accuracy of 96.9% and load weight accuracy of 93.1%. Finally, in a follow-up to their previous study, Lim and D’Souza [32] analyzed gender differences in classification results and found that gender-specific classifiers could improve load weight classification accuracy over non-gender-specific ones by approximately 5%.

In the current study, we sought to build on this previous work by using a large number of IMUs and EMG sensors together with multiple different classifiers to study multiple classification problems involving different load positions and different symmetric and asymmetric (more load in one hand) loads. Our goal was to identify the most important sensors and most useful signal processing and pattern recognition algorithms for different classification problems, which can help guide the development and optimization of future assistive devices for lifting and carrying. We consider our study to be a proof of concept that shows the feasibility of performing such classification, though we acknowledge that further work on practical topics such as sensor drift is necessary before the algorithms can be used in real-world devices.

The paper is organized as follows: The Section 2 consists of three subsections describing the hardware, study protocol, and data analysis (feature extraction and classification). The Section 3 then presents classification accuracies and top selected features for different classification problems. Finally, the Section 4 first discusses the results, then describes major study limitations, generalizability to other IMU systems, and ways in which the results of the work could be used in applied settings.

## 2. Materials and Methods

### 2.1. Hardware

The Xsens MVN Link (Xsens Technologies BV, Enschede, Netherlands), a commercially available inertial measurement unit (IMU) system was used for the study. It consists of 17 IMUs that are attached to the feet, lower legs, upper legs, pelvis, shoulders, sternum, head, upper arms, forearms, and hands for full-body motion capture. Each unit consists of 3D linear accelerometers, 3D rate gyroscopes, 3D magnetometers, and a barometer. Full specifications can be found in the MVN User Manual [33], but the basic sensor performance is characterized as follows: static accuracies for roll/pitch and heading are 0.2 and 0.5 degrees, respectively; dynamic accuracy is 1 degree root-mean-square; accelerometer range is ±16 g; and gyroscope range is ±2000 degrees/s.

**Figure 1:**
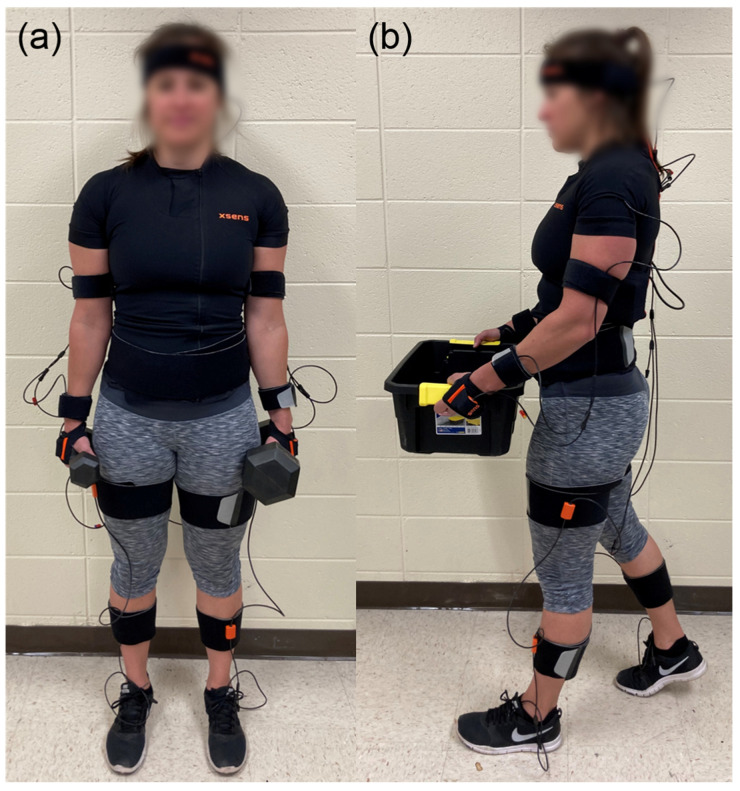
A participant carrying asymmetric dumbbells (**a**) or a box (**b**) while wearing the full-body inertial measurement system (Xsens) and wireless electromyography electrodes on the low back (not visible, obscured by clothes).

All trunk IMUs are placed inside a special shirt with pockets for the sensors as well as a battery and receiver pocket on the back: the sternum sensor is positioned flat on the chest, the pelvis sensor flat on the sacrum, and the shoulder sensors are placed on shoulder blades. A special headband is used to attach one sensor to the back of the head. The upper arm sensor is attached with a strap on the lateral side above the elbow while the forearm sensor is attached with a strap on the lateral and flat side of the wrist. Fingerless gloves are provided with a pocket for the hand sensor on the back side of the hand. The upper leg sensor is placed on the lateral side above the knee while the lower leg sensor is placed on the medial surface, flat on the shin bone. The two foot sensors are secured to shoes using foot pads on the middle of the bridge of the foot. A photo of a participant wearing the sensors is shown in Figure 1; a more detailed video tutorial demonstrating the exact sensor placement is available on the Xsens Technologies website.

Data from each IMU are transmitted to a computer wirelessly at 240 Hz. There, the Xsens MVN Fusion Engine software combines the data from the individual units with a biomechanical model of the subject’s body to obtain segment positions and orientations [33]. The MVN biomechanical human model has 23 segments with 22 joints. The Fusion Engine output is a full kinematic description of each segment, which includes position, velocity, acceleration, orientation, angular velocity, and angular acceleration. Therefore, the Xsens motion capture system enables full body pose reconstruction comparable to optical systems, with about a 1% error in segment traveled distance; soft issue artifacts are minimized to ~2 degrees root-mean-square [33].

To evaluate muscle demand, the electromyogram (EMG) of the left and right erector spinae was measured using a Trigno Avanti System (Delsys Inc., Natick, MA, USA) with a sampling frequency of 2148 Hz. Two wireless bipolar electrodes were placed at L3 height, approximately 4 cm left and right from the midline of the spine. Two pilot tests were done with additional electrodes on the rectus abdominis and external obliques, but showed no major changes and were therefore not used for the full study.

### 2.2. Study Protocol

The study was conducted in the Biomechanics Laboratory of the University of Wyoming. The study was conducted in accordance with the Declaration of Helsinki, and the protocol was approved by the Institutional Review Board of the University of Wyoming (protocol #20200129DN02643). Ten healthy, injury-free participants were recruited for the study (4 female; 24.0 ± 1.6 years old, 175.6 ± 11.1 cm, 72.5 ± 13.0 kg, all right hand dominant). The protocol was first briefly described, and participants signed an informed consent form. Participants were asked to wear athletic shoes and to avoid very loose clothing that would interfere with the sensor straps. Prior to attaching the sensors, each participant’s body dimensions (height, foot length, arm span, ankle height, hip height, hip width, knee height, shoulder width, shoulder height, and shoe sole height) and weight were measured. A simple test of muscle activation was performed to make sure the electrodes were attached adequately, and an Xsens calibration routine was carried out to account for sensor position/orientation and body shape variance. To carry out the calibration, participants’ dimensions were first entered into the Fusion Engine software. Next, participants were asked to hold a specific static pose for a few seconds, then walk naturally a few steps forward, turn around and walk back to the starting place and hold the same static pose. The Fusion Engine determined the orientation and position of the sensors relative to their segments during this process.

Participants were then instructed to pick a set of dumbbells or a weighted box. They were asked to stand still with the load (dumbbell(s) on the side of the body with straight arms or box in front of the body at waist level) until they received a verbal “start” signal from the experimenter. They then carried the load at their preferred pace in a straight line across the laboratory with head facing forward, stopped on the other end, and stood still until a verbal stop signal was given. This single walk across the lab was considered an individual ‘trial’. Each participant completed 19 trials:

- one control trial of walking without any load,
- three trials with a 30-lb box (18 cm height × 32 cm width × 45.5 cm length):
  o one trial symmetrically loaded with 15 lb on each side of the box,
  o one trial with 5 lb on the left side of the box and 25 lb on the right,
  o one trial with 25 lb on the left and 5 lb on the right,
- fifteen trials with different sets of dumbbells:
  o six trials with one dumbbell: 5, 15, and 25 lb in only left or only right hand,
  o three symmetric pairs of dumbbells: 5, 15, and 25 lb in each hand,
  o six trials with asymmetrically paired dumbbells: 5 lb in one hand and 15 lb in the other and vice versa, 15 lb and 25 lb dumbbells, and 5 and 25 lb dumbbells.

These loads were chosen based on prior studies of load position and weight, which similarly studied no load, front load, and symmetric/asymmetric side loads [24,29,31] and used similar weights [24,26,27,32]. Participants were allowed to rest between trials as needed, and the order of trials was randomized between participants. After each trial, participants returned to the starting point and picked up the next load without data being collected; thus, all trials involved walking in the same direction. An example of a participant equipped with sensors while carrying loads is shown in Figure 1.

Lastly, a maximum voluntary contraction (MVC) test of the erector spinae muscles was done by having the participant lie on their stomach and try to lift their upper body (without using their arms) as much as possible for 5 s while an experimenter pushed down on the participant’s shoulders with a steady force.

### 2.3. Data Analysis

#### 2.3.1. Feature Extraction

Each trial was first segmented into individual steps based on heel-strike timestamps obtained from the Xsens MVN Fusion Engine. The algorithm used to detect heel-strike is proprietary and not shared by Xsens Technologies, but is based on detecting vibration patterns caused by the foot hitting the ground, then verifying that the foot does not move after the vibration pattern (since it is in stance phase). The timestamps were used to segment the signals into right steps (from left heel strike to right heel strike) and left steps (from right heel strike to left heel strike). Multiple features (described below) were then calculated for each step, and the mean value of each feature was taken over all left steps and over all right steps separately to obtain the left and right feature for that trial.

The Xsens inertial system output 66 total signals: angles for 22 joints in 3 planes. Four different features (mean and variance of the raw signals and mean and variance of the absolute values of the signals) were calculated from all 66 signals, resulting in 264 features for each step and 528 inertial features total for each trial (due to averaging separately over left and right steps).

EMG signals were detrended, filtered with a second-order 20–450 Hz bandpass filter and rectified. The EMG envelope was extracted by applying a 2nd order Butterworth lowpass filter with a cutoff frequency of 10 Hz. Each envelope was normalized by the maximum value measured during that participant’s MVC test. Separately for the left and right erector spinae, the mean and RMS were calculated for all left and all right steps, resulting in 8 features (2 muscles × 2 feature types × 2 left/right steps). These steps (including choice of cutoff frequencies) represent a standard low-level EMG analysis approach in the literature [15]. Peak detection was then performed on the unsegmented normalized signals (minimum peak amplitude 10% of MVC; minimum peak duration 0.2 s), and the median amplitudes of all the peaks in each trial from the left and right muscle were used as the last two features; these were the only two features that were not calculated separately for left and right steps.

For any trial where either Xsens or EMG data (but not both) were lost, the features from that sensor system were all set to zero.

#### 2.3.2. Classification

Five classification problems were defined for the collected dataset. There was one two-class classification problem:

- box vs. no box trials, done with the entire dataset (with “no box” consisting of trials with dumbbells or no load).
- There were four three-class classification problems:
- box (3 trials/person) vs. 1 dumbbell (6 trials) vs. 2 dumbbells (6 trials); the ‘no load’ trial was skipped,
- symmetric load (4 trials, including ‘no load’) vs. <20 lb difference between left- and right-side load (8 trials) vs. ≥20 lb difference between left- and right-side load (4 trials); the three box trials were skipped,
- symmetric load (4 trials, including ‘no load’) vs. ≤10 lb difference between left- and right-side load (6 trials) vs. >10 lb difference between left- and right-side load (6 trials); the three box trials were skipped,
- symmetric load (4 trials, including ‘no load’) vs. heavier left-side load (6 trials) vs. heavier right-side load (6 trials); the three box trials were skipped.

Each classification problem was analyzed with three different input feature sets:

- only the 528 inertial features,
- only the 10 EMG features,
- all 538 inertial and EMG features.

For each input feature set, sequential floating forward selection [34] was first used to find the most informative set of features. Starting from an empty feature set, the method sequentially added the feature with the lowest *p*-value to the set (with regard to the ability to discriminate between classes, taking into account the contribution of features already in the set) as long as that feature met an inclusion threshold. After each inclusion step, it also removed individual features from the set if they met an exclusion threshold. The initial thresholds were *p* < 0.05 for inclusion and *p* > 0.05 for exclusion; however, if the final selected set had more than 50 features in it, the threshold was reduced from 0.05 to 0.01 and the process was re-run; if the second selected set still had more than 50 features, the threshold was finally reduced to 0.001 (only done in the case of box vs. no box). This was done to reduce overfitting.

After completion of feature selection, four different classifiers were trained for each reduced data set: linear discriminant analysis (LDA), ensemble decision tree (EDT), support vector machine (SVM) with a linear kernel, and multiple linear regression (REG) [35,36]. They were implemented using prebuilt functions in the pattern recognition toolbox of MATLAB 2019a (MathWorks, Natick, MA, USA). To use multiple linear regression as a classifier, its continuous output value was rounded to the closest class.

The classifiers were evaluated using leave-participant-out cross-validation: 9 participants’ data were used to train, and one participant’s data were used to test the classifier. This was repeated as many times as there were participants, with each participant serving as the test participant once. The mean classification accuracy (percentage of correctly classified trials) over all 10 test participants was used as the overall accuracy of that classifier.

## 3. Results

**Figure 2:**
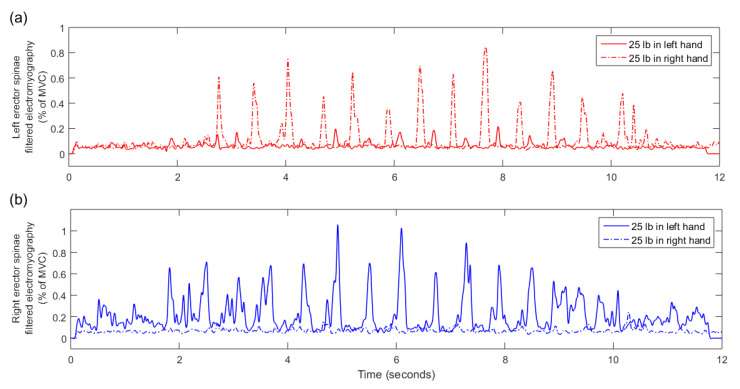
An example of one participant’s rectified and bandpass-filtered electromyograms of the left (**a**) and right (**b**) erector spinae muscle for asymmetric (unilateral) dumbbell carrying. The full line shows muscle activity for a trial where the participant carried a 25-lb dumbbell in their left hand while the dotted line shows muscle activity for a trial with a 25-lb dumbbell in their right hand. A common, though subjectively observed, pattern was that the left muscle (**a**) exhibited higher electromyogram values when a dumbbell was held in the right hand, and vice versa. MVC—peak value obtained during maximum voluntary contraction.

**Figure 3:**
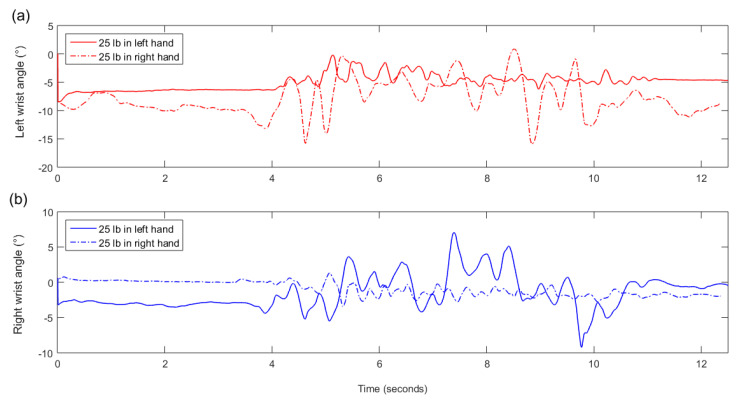
An example of one participant’s left (**a**) and right (**b**) wrist pronation/supination angle during asymmetric (unilateral) dumbbell carrying. The full line shows the angle for a trial where the participant carried a 25-lb dumbbell in their left hand while the dotted line shows the angle for a trial with a 25-lb dumbbell in their right hand. A common, though subjectively observed, pattern was that there is less wrist angle variability when a hand is holding a dumbbell—the variability in the left wrist angle (**a**) is lower when the dumbbell is held in the left hand, and the variability in the right wrist angle (**b**) is lower when the dumbbell is held in the right hand.

Raw data from all participants and trials are available in the Supplementary Material. EMG data for one asymmetric box trial of one participant were lost. All EMG features for that trial were set to zero. There were no missing inertial data for any participant. A typical example of an EMG signal for an asymmetric (unilateral) 25 lb dumbbell carrying trial is presented in Figure 2 while a typical example of a wrist pronation/supination angle signal is presented in Figure 3.

Looking at results of the four classification algorithms, the REG classifier exhibited the overall highest classification accuracies for inertial data and for the combination of inertial and EMG data. On the other hand, the SVM classifier exhibited the highest accuracies when only EMG data were used. Thus, to save space, Table 1 presents results of the REG classifier for inertial data alone, results of the SVM classifier for EMG data alone, and results of the REG classifier for the combination of inertial and EMG data. In all five classification problems, the combination of inertial and EMG data yielded the highest accuracy.

The top 5 features selected by sequential floating forward selection for all five classification problems were all from inertial data, as shown in Table 2. Data from seven joints never appeared among the top 5 selected features: T9-T8, C1-head, right hip, right knee, right ankle, right and left ball of the foot. Nine of the 25 features were calculated from right steps while 16 were calculated from left steps. The most commonly used raw signals for calculation of the top 5 features were the wrist angles.

## 4. Discussion

IMUs alone were able to very accurately differentiate between box vs. no box (dumbbells or nothing) trials (100% accuracy) and between box vs. one dumbbell vs. two dumbbells (96.1% accuracy). The 100% accuracy for box vs. no box is not surprising, as holding a box requires both arms to be raised in front of the body while no box requires the arms to be held at the side of the body—two very different postures. The slightly lower accuracy for the box vs. one dumbbell vs. two dumbbells problem is also expected, as this is a slightly more difficult version of the box vs. no box problem. The main increased challenge is in differentiating between one and two dumbbells, which is achievable using only inertial data—when not holding a dumbbell, the participant’s arm tended to exhibit more motion during gait, as seen in Figure 3. This is also evidenced by the top selected features, which were all from IMUs on the arms.

A lower accuracy is obtained when classifying between symmetric and different asymmetric dumbbell loads. This represents a greater challenge, as different dumbbell weights do not affect kinematics as much as the presence or absence of a dumbbell. In all three classification problems, including EMG increased classification accuracy by 6–8% (e.g., in the 10 lb case: 70.6% with only IMUs and 78.8% with both IMUs and EMG); as seen in Figure 2, asymmetric loads result in very asymmetric EMG of the left and right erector spinae. Interestingly, even though including EMG increased classification accuracy, the top 5 selected features were still all from IMUs (Table 2); the reason for this is currently unclear, though EMG features appeared as sixth and/or seventh selected features in both cases. The selected features in these cases include data from IMUs all over the body, which may indicate changes in overall body balance as a result of asymmetric load; however, it may also be statistical noise due to the relatively small dataset.

Overall, from the top selected features (Table 2), we can see that not all sensors are required for accurate classification. For example, neither the head IMU nor any of the IMUs on the right leg appear in Table 2, and a follow-up analysis found that removing all features from these IMUs prior to classification resulted in negligible changes in classification accuracy. Thus, many of the IMUs could be removed for a practical system. Additional simplifications could be made depending on the expected loads; for example, if participants in a real-world application are expected to mainly deal with symmetric loads, IMUs from one arm could potentially be omitted.

Finally, based on the results (low contribution of EMG), we were concerned that the EMG analysis may have been suboptimal: the EMG was segmented into steps, and the initial and final parts of each trial (i.e., gait initiation and termination) were included in the analysis. As an alternative approach, we thus performed a follow-up analysis where we manually extracted the steady-state section of each trial based on visual analysis and calculated features across the entire steady-state section as well as across all signal peaks in the steady-state section. However, these alternative features did not improve classification accuracy, and this follow-up analysis is thus not described in detail. In a second follow-up analysis, we also investigated whether IMU-based classification accuracy would increase if skewness and kurtosis were also calculated and added to the feature set; however, this also did not improve classification accuracy.

### 4.1. Study Limitations

Perhaps the main limitation of the study was that EMG sensors were placed only on the erector spinae. This placement was chosen based on our previous study of lifting activities with a trunk exoskeleton, which showed significant changes in the EMG of the erector spinae and rectus abdominis [5]; the rectus abdominis was removed from this study after it was not found to be informative in a pilot measurement. However, for load position and weight classification, EMG sensors should likely have also been placed on the arms.

Furthermore, the study only included a few different weights, which were not tailored to individual participants’ abilities; classification into, e.g., loads that are easy vs. difficult to lift for a specific participant may have resulted in very different results. The study also only included one frontal box position as opposed to, e.g., holding a box low or high in front of the body, and did not include any back load, commonly seen in other studies of carrying behavior [23,26,27]. All participants also walked at a self-selected walking speed, which may not have adequately captured differences between slow and fast gait. Nonetheless, while additional load positions, weights, and gait speeds might change the accuracy and the relative contribution of different sensors, the same feature extraction and classification approaches could be used in such scenarios as well with minimal modification.

Additionally, the feature selection process only focused on increasing accuracy rather than ensuring that the selected features represented a meaningful set. For example, the top selected features for the box vs. no box problem include left wrist pronation/supination on left steps and on right steps as two separate features (indicating possible redundancy from a modeling perspective), and features were calculated separately from raw and absolute joint angles even though those are simply two versions of the same signal. The size of the selected feature set was also sometimes larger than would be ideal for robustness (up to 45 features), and the top selected features were very different between the 20 lb and 10 lb classification cases even though these are similar practical problems. Future work may investigate, for example, classification models based on fundamental biomechanical knowledge, which may prove to be simpler, more interpretable and/or more robust.

Finally, our study included only right-handed participants. While we cannot be certain, it is possible that left-handed participants may exhibit different behaviors that affect overall accuracy or the contribution of different features. For example, we observed that data from four joints on the right side of the body and only one joint on the left side never appeared among top features, and that features from left steps were more common among top features than features from right steps; part of this pattern may be reversed for left-handed participants. To ensure robust classification, future studies should include at least some left-handed participants.

### 4.2. Generalizability to Other Sensor Technologies

The study was conducted with commercially available IMU and EMG sensors. We believe that results obtained with our EMG sensors could be easily applied to other EMG sensors, which are generally similar and do not require advanced signal processing. The Xsens IMU system, however, is relatively expensive and handles major aspects of IMU signal processing: sensor calibration, IMU orientation calculation from raw measurements, and calculation of human joint angles from individual IMU orientations. While many cheaper IMU technologies and many software methods for IMU sensor fusion exist, they are likely to be less accurate—for example, due to greater sensor drift over time or during rapid movements. Thus, the study should ideally be repeated with a cheaper system to identify the degree to which a decrease in IMU accuracy may affect classification results.

### 4.3. Application to Assistive Devices and Occupational Monitoring

The most common control systems for assistive devices such as orthoses and prostheses have three main components [7,14,15]: they must identify the current behavior (e.g., standing still vs. walking on level ground vs. walking on stairs), they must rapidly detect transitions between behaviors (e.g., sit-to-stand, picking up an object), and they must provide appropriate assistance for the current behavior. In our previous work, we detected the beginning and end of a lift [21], which could be used to detect the transition to/from carrying behavior, and the type of carrying behavior could then be detected using the algorithms proposed in this paper. To properly assist the user, an assistive device such as a trunk exoskeleton could then switch to a control algorithm suited to the current load placement and weight; different active control strategies for such exoskeletons have already been proposed in the literature [4]. Such intelligent behavior switching has the potential to provide more personalized, effective assistance than simply providing the same assistance regardless of load. The wearable sensors used in this study could even be combined with sensors installed into the assistive device to further increase classification accuracy and assistance effectiveness.

In addition to control of assistive devices, the algorithms developed in this work could enable more convenient and efficient monitoring of workers in physically demanding occupations such as manual materials handling. Workers could wear the sensors for hours at a time, and the developed algorithms could automatically identify periods of potentially risky behavior (e.g., prolonged carrying of heavy loads in an unergonomic position), allowing supervisors to make adjustments to the workplace or an individual worker’s schedule. While this could already be done with manual observation or manual analysis of sensor data, automated classification would greatly speed up the process.

Nonetheless, despite promising applications, we acknowledge that our study is a proof-of-concept one, and that practical issues such as IMU drift over time (see Section 4.2) need to be addressed before more applied work can be conducted on this topic.

## 5. Conclusions

We developed and evaluated classification algorithms to differentiate between different loads (frontal and symmetric/asymmetric side loads) during gait based on a combination of IMUs and EMG. The algorithms were able to differentiate a box and other loads (side/no load) with an accuracy of 100%, between a box vs. unilateral side load vs. bilateral side load with an accuracy of 96.1%, and between different asymmetry levels with accuracies of 75–79%. When differentiating between a box vs. unilateral vs. bilateral side loads, the most informative sensors were IMUs on the arms; when different asymmetry levels were analyzed, IMUs on the trunk and leg also provided useful information. While the study is limited by a lack of EMG sensors on the arms and the use of a limited number of load positions/weights, it does indicate that wearable sensors can be used to differentiate between different load positions and weights during carrying with a high degree of accuracy. In the future, such wearable sensors and classification algorithms could be used to control assistive devices such as trunk exoskeletons, or to assist with long-term monitoring of workers in physically demanding occupations.

## Figures and Tables

**Table 1:** Number of selected features and classification accuracies for all five classification problems using three input data types: only inertial, only electromyography (EMG), or inertial + EMG. In each field, the first value is the number of selected features while the second is classification accuracy. Results are given for the support vector machine-based classifier in case of EMG data alone and for the regression-based classifier in the cases of inertial data alone and the combination of inertial and EMG data, as these classifiers exhibited the highest accuracies for those data types.

Classes	Inertial	EMG	Inertial + EMG
box vs. no box	9, 100%	5, 90.5%	9, 100%
box vs. 1 dumbbell vs. 2 dumbbells	21, 96.1%	4, 57.2%	21, 96.1%
symmetric vs. <20 lb difference vs. ≥20 lb difference between left and right side	32, 71.9%	3, 50.0%	42, 77.5%
symmetric vs. <10 lb difference vs. ≥10 lb difference between left and right side	39, 70.6%	10, 63.7%	45, 78.8%
symmetric vs. heavier left vs. heavier right	16, 67.5%	6, 52.5%	33, 75.0%

**Table 2:** Top 5 selected features for each classification problem. R—right; L—left; var—variance, abs—absolute value.

Classification Problem	Selected Features
box vs. no box	mean (R elbow flexion/extension) on L steps
	mean (L wrist pronation/supination) on L steps
	mean (abs (R wrist pronation/supination) on L steps
	mean (abs (L elbow flexion/extension)) on L steps
	mean (abs (L wrist pronation/supination)) on R steps
box vs. 1 dumbbell vs. 2 dumbbells	mean (R elbow flexion/extension) on L steps
	mean (abs (R shoulder flexion/extension)) on L steps
	var (abs (L elbow ulnar/radial deviation)) on L steps
	mean (R wrist ulnar/radial deviation) on L steps
	mean (abs (L wrist ulnar/radial deviation)) on R steps
symmetric vs. <20 lb difference vs. ≥20 lb difference between left and right side	var (abs (L ankle flexion/extension)) on R steps
	mean (abs (L knee internal/external rotation)) on R steps
	var (L4-L3 axial bending) on L steps
	var (L ankle internal/external rotation) on L steps
	var (R wrist pronation/supination) on R steps
symmetric vs. < 10 lb difference vs. ≥ 10 lb difference between left and right side	var (abs (L1-T12 axial bending)) on R steps
	mean (L shoulder internal/external rotation) on R steps
	var (L1-T12 axial bending) on L steps
	mean (abs (L hip internal/external rotation)) on R steps
	mean (L T4-shoulder abduction/adduction) on L steps
symmetric vs. heavier left vs. heavier right	mean (abs (T1-C7 lateral bending)) on L steps
	var (abs (L5-S1 axial bending)) on R steps
	mean (R T4-shoulder flexion/extension) on steps L
	var (L ankle abduction/adduction) on L steps
	mean (abs (R T4-shoulder internal/external rotation)) on L steps

## Supplementary Materials

The data collected for this study are available at https://doi.org/10.5281/zenodo.3941377.

## Abbreviations

The following abbreviations are used in this manuscript:

DB	Dumbbell
EMG	Electromyograph
ES	Erector spinae
IMU	Inertial measurement unit
L	Left
LDA	Linear discriminant analysis
R	Right
RMS	Root-mean-square
SVM	Support vector machine
